# ^68^Ga-DOTATATE PET-CT as a tool for radiation planning and evaluating treatment responses in the clinical management of meningiomas

**DOI:** 10.1186/s13014-021-01875-6

**Published:** 2021-08-16

**Authors:** Emily S. Kowalski, Rahul Khairnar, Anton A. Gryaznov, Vivek Kesari, Antony Koroulakis, Prashant Raghavan, Wengen Chen, Graeme Woodworth, Mark Mishra

**Affiliations:** 1 Department of Radiation Oncology, University of Maryland Medical Center, Baltimore, MD USA; 2 Department of Pharmaceuticals Health Services Research, University of Maryland School of Pharmacy, Baltimore, MD USA; 3 Department of Radiology, University of Maryland School of Medicine, Baltimore, MD USA; 4 Department of Nuclear Medicine, University of Maryland School of Medicine, Baltimore, MD USA; 5 Department of Neurosurgery, University of Maryland School of Medicine, Baltimore, MD USA; 6 Department of Radiation Oncology, University of Maryland School of Medicine, Baltimore, MD USA

## Abstract

**Background and purpose:**

Meningiomas express the somatostatin receptor (SSTR), which normal bone and brain lack. PET imaging with SSTR ligands such as ^68^ Ga-DOTATATE have been recently shown to aid in the imaging and identification of menginiomas. We hypothesize that ^68^ Ga-DOTATATE PET/CT in conjunction with MRI aids in radiation (RT) target volume delineation and evaluating treatment response.

**Materials and methods:**

Nineteen patients with meningiomas underwent ^68^ Ga-DOTATATE PET/CT and MRI for RT planning and/or post-treatment follow-up. Meningiomas were grade I (n = 9) or not biopsied (n = 8) and frequently involved base of skull (n = 10). Ten (53%) patients received post-operative RT and 9 (47%) received fractaionted RT. In the subgroup that underwent both pre- and post-RT ^68^ Ga-DOTATATE PET as well as MRI (n = 10), ROVER (ABX GmbH, Radeberg, Germany) adaptive thresholding software was utilized to measure total lesion activity (mean and max) before and after treatment. Tumor volume based on MRI was calculated before and after treatment. Total lesion activity and tumor volume changes were compared using Wilcoxon signed rank test.

**Results:**

^68^ Ga-DOTATATE PET/CT identified intraosseous (n = 4, 22%), falcine (n = 5, 26%) and satellite lesions (n = 3, 19%) and clarified the diagnosis of meningioma, resulting in a change in management in three patients. Mean total lesion activity decreased 14.7% (median), from pre to post-RT ^68^ Ga-DOTATATE PET [range 97–8.5% (25–75%),S = − 26.5, *p* = 0.0039]. Max total lesion activity decreased 36% (median) over the same period [range 105–15% (25–75%), S = − 26.5 *p* = 0.0039]. In contrast, meningioma volumes based on MRI measurements did not significantly change per RECIST criteria and Wilcoxon signed rank test (S = − 3, *p* = 0.7422).

**Conclusion:**

^68^ Ga-DOTATATE PET/CT helped confirm suspected diagnoses and delineate target volumes particularly when lesions involved osseous structures and the falx. Mean and max total tumor ^68^ Ga-DOTATATE activity on PET/CT decreased at three months following RT despite stable tumor volumes on MRI. Future studies are warranted to (1) assess the sensitivity and specificity of ^68^ Ga-DOTATATE PET/CT, (2) evaluate the impact of ^68^ Ga-DOTATATE PET/CT-based planning on treatment outcomes, and (3) assess the prognostic significance of these post-treatment imaging changes.

## Introduction

Meningiomas are the most common primary brain tumor with a reported incidence of 8.33 per 100,000 [1] Radiation therapy (RT) is the preferred treatment for symptomatic meningiomas not amenable to surgical resection and is delivered in the adjuvant setting following resection of WHO II and III meningiomas.

Advanced RT techniques, including pencil beam scanning proton therapy (PBSPT), stereotactic radiosurgery (SRS), and intensity modulated radiotherapy (IMRT) are utilized to deliver a highly conformal dose to tumor, while also reducing radiation dose to surrounding healthy brain tissue. The efficacy of these techniques is highly dependent on accurate target delineation, which is currently guided by contrast-enhanced MRI.

The majority of meningiomas are now known to highly express somatostatin receptor-2 (SSTR2) [2, 3]. SSTR2 receptors have been exploited to allow for precise diagnostic and functional imaging of meningiomas. Klutman et al. demonstrated that Indium111 octreotide SPECT better identified residual or recurrent tumor versus MRI in post-operative surveillance of meningiomas [4]. More recently, PET utilizing radiotracer ligands of SSTR2 with ^68^ Ga-DOTA have gained traction and are now used frequently in Europe in the diagnosis and treatment planning for meningiomas.

^68^ Ga-DOTATATE PET/CT provides high contrast images of meningiomas, given that the surrounding normal brain and bone tissues lack the SSTR2. Furthermore, it has been shown to be more sensitive than MRI in detecting meningiomas [5, 6]. ^68^ Ga-DOTATATE PET/CT is a particularly useful diagnostic tool when the risk of biopsy is prohibitive, as in the case of suspected optic nerve meningiomas [7]. Previous studies have also examined the utility of ^68^ Ga-DOTATATE PET/CT for contouring and RT target delineation and demonstrated that it often results in modified treatment volumes compared to those created based upon CT and MRI [8–12]. However, post-treatment changes with ^68^ Ga -DOTATATE PET/CT following radiation therapy have not been previously described.

In this series we report our experience incorporating ^68^ Ga -DOTATATE PET/CT alongside conventional imaging in the workup, planning and post-treatment follow-up imaging assessment of patients with meningiomas treated with conventionally fractionated radiotherapy. Additionally, for those patients with both pre and post-treatment ^68^ Ga -DOTATATE PET/CT we quantify the change in mean and max total tumor ^68^ Ga-DOTATATE activity as compared to the change in tumor volume on pre and post-treatment MRI.

## Methods and materials

### Patients

We conducted an institutional review board approved retrospective evaluation of nineteen patients with clinically symptomatic meningiomas treated with radiation therapy at a single academic institution from 2017 to 2019. Patients underwent PET/CT with ^68^ Ga-DOTATATE radiotracer, CT and brain MRI for radiation planning (n = 18, 95%) and/or post-treatment follow-up (n = 10, 53%). Inability to secure insurance authorization was a primary reason a portion of our population did not receive both pre and post treatment follow up ^68^ Ga-DOTATATE PET/CT. Intracranial meningiomas were diagnosed by biopsy or based on classic imaging findings and multi-disciplinary tumor-board consensus opinion. Ten patients (53%) underwent a prior sub-total resection followed by adjuvant radiation, nine patients (47%) received radiation treatment alone. Medical records were reviewed and key clinical attributes were recorded.

### Imaging

#### Conventional CT simulation with MRI fusion

Patients were immobilized in the RT treatment position using a three-point thermoplastic mask and thin-sliced CT scans were performed for RT treatment planning. Brain MRIs were performed within a week of treatment planning, and were fused to the RT CT-simulation images for target delineation.

#### 68GA-DOTATATE PET/CT image acquisition

Brain ^68^ Ga-DOTATATE PET/CT images were acquired on an integrated PET/CT scanner (Biograph mCT, Siemens) 60 min after intravenous administration of approximate 5 mCi of ^68^ Ga-DOTATATE. Images were reconstructed in the sagittal, transverse and coronal axes for evaluation. A low dose CT scan was performed for attenuation correction.

#### Radiotherapy details

Initially, gross tumor volume (GTV) was delineated based on both the MRI and CT dataset. ^68^ Ga-DOTATATE PET/CT was subsequently fused with CT simulation images, and the GTV was modified if there were PET-avid regions identified on PET imaging that were not previously identified on the MRI, but were felt to represent meningioma after review by the radiation oncologist and neuroradiologist. In accordance with our institutional clinical practice guidelines, a 3–5 mm clinical target volume expansion was added to the GTV for WHO grade I tumors. This margin was reduced to 0 mm around natural barriers and expanded at physician discretion when tumor infiltration into surrounding brain structures was less certain. The majority of patients were treated with fractionated PBSPT (n = 12, 63%) to a median dose of 5220 cGy in 29 fractions (range 4500–5940 cGy). Seven patients (37%) were treated with fractionated volumetric arc modulated radiotherapy to a median dose of 5220 cGy in 29 fractions (range 5220–5400 cGy).

#### Follow-up 68GA-DOTATATE PET/CT

A subgroup of ten patients received a follow-up ^68^ Ga-DOTATATE PET/CT and independent MRI (n = 10) or PET-MR (n = 2) 3 months following completion of radiotherapy.

### Post-RT analysis

#### PET/CT image analysis

Two nuclear medicine physicians, blinded to the patients’ medical history, independently evaluated pre and post radiation PET/CT images using an adaptive threshholding system (ROVER software; ABX GmbH, Radeberg, Germany). DICOM images were obtained, spherical or cylindrical masks were placed over an active lesion and the adaptive threshholding algorithm of ROVER auto segmented each lesion’s boundaries. The software utilized a threshold of 50% SUV max. ROVER automatically determined the lesion volume and quantitative PET parameters including SUVmax, SUVmean, and partial volume corrected (PVC) SUVmax and SUVmean for the treated lesions. Total lesion activity (TLA) was calculated by multiplying the PVC SUV (mean or max) and the dotatate volume determined by ROVER.

#### MRI image analysis

MRIs were reviewed by a neuroradiologist and the treating radiation oncologist to document imaging indications of response to radiotherapy. RECIST criteria was utilized to objectify treatment response on 3 month follow-up MRI. The longest diameter of the lesion was measured on pre- and post-treatment MRI, and the percent change from pre-treatment was calculated. Threshholds for response were consistent with RECIST criteria and designated as follows: 1. Complete response was indicated with resolution of all enhancing tumor at 3 months; 2. Partial response was noted if ≥ 30% decrease in maximal diameter at 3 months; 3. Progressive disease was noted if ≥ 20% increase in the sum of max diameter at 3 months; 4. Stable disease was noted for all other responses [13].

#### Clinical analysis

Patients were seen in follow-up 3 months after treatment by the treating radiation oncologist. Clinical improvement was recorded if patients reported reduction in their presenting symptoms or the physician assessed improved neurologic function on physical exam.

#### Statistics

A wilcoxon signed-rank test was performed to determine the difference in total lesion activity between each patient’s pre- and post-treatment ^68^ Ga -DOTATATE PET/CT and the difference in volume between their pre- and post-treatment MRIs at the same timepoints. Signed-rank test was utilized because the two groups are dependent (pre- and post- radiotherapy imaging assessment of the same sample) and do not follow a normal distribution.

## Results

The median age of the population was 65 years and most patients were female (n = 16, 84%). For the subset of patients who underwent pre- and post-treatment ^68^ Ga-DOTATATE PET/CT. Treated meningiomas were located at the base of skull (n = 4, 40%), falx (n = 3, 30%), frontoparietal cerebral convexities (n = 1, 10%), cerebellar tentorium (n = 1, 10%) and optic nerve sheath (n = 1, 10%). Two patients had recurrent tumors. One patient received prior radiosurgery (17 Gy). Six patients underwent biopsy for WHO grade 1 (n = 5, 50%) or WHO grade 2 (n = 1, 10%) tumors. Patient and tumor characteristics are summarized in Table 1.

**Table 1:** Patient characteristics

Patient number	Tumor location^a^	Recurrent (Yes/No)	Prior resection (Yes/No)	Prior RT (Yes/No)	Dose (Gy)	RT Modality	PET identified area not well seen on MRI
*Patients with pre and post radiation 68 Ga-DOTATATE PET/CT and MRI*							
1	1	No	No	No	52.2	Proton	Osseous involvement
3	1	No	No	No	52.2	Proton	
4	1	No	Yes	No	54.0	VMAT	Residual tumor post-resection
12	1	No	Yes	No	52.2	Proton	Extent of falx involvement
6	2	No	Yes	No	54.0	VMAT	Extent of falx involvement
7	2	No	No	No	54.0	Proton	Extent of falx involvement
8	2	Yes	Yes	Yes	59.4	Proton	Extent of falx involvement
9	3	Yes	Yes	No	54.0	Proton	Osseous involvement
10	4	No	No	No	50.4	Proton	Optic nerve
11	5	No	Yes	No	52.2	Proton	
*Patients with 68 Ga-DOTATATE PET/CT used for radiation planning*							
2	1	No	No	No	52.2	VMAT	Osseous involvement
5	1	Yes	Yes	No	52.2	VMAT	Osseous involvement
13	1	Yes	Yes	No	52.2	VMAT	Osseous involvement
14	1	No	No	No	52.2	VMAT	Extent of falx involvement
15	1	Yes	No	Yes	45.0	Proton	Additional meningioma lesion confirmed
16	1	Yes	Yes	Yes	3.0	Proton	Recurrent meningioma
17	5	No	No	No	18.0	Stereotactic Radiosurgery	Ruled out meningioma and treated as brain metastasis
18	5	No	No	No	52.2	Proton	
19	3	Yes	Yes	No	59.4	Proton	Osseous involvement

^a^Tumor location: 1. Base of skull; 2. Falx; 3. Brain convexity; 4. Optic nerve; 5. Cerebellar pontine angle/ posterior fossa

### Pre-treament imaging

**Fig. 1:**
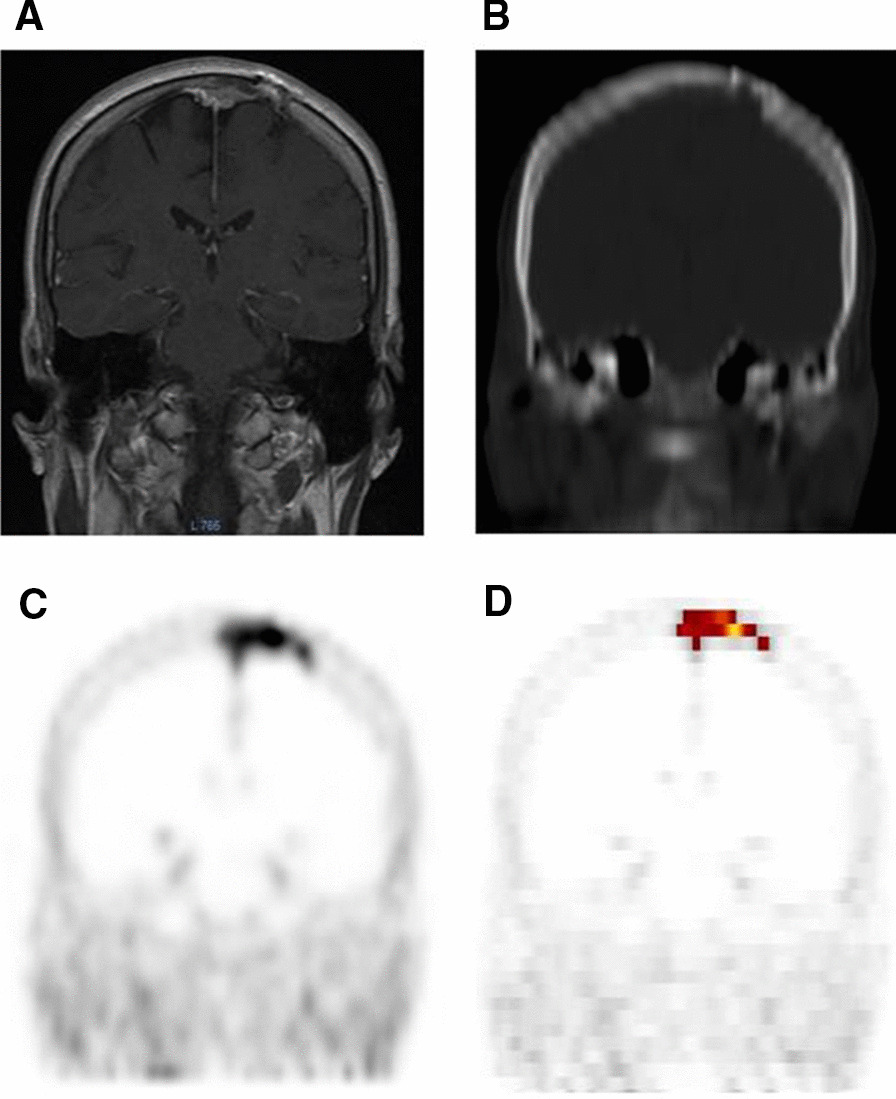
Left parietal meningioma with calvarial involvement. **A** Pre-treatment MRI, **B** Pre-treatment CT-scan, **C**. Pre-treatment 68 Ga-DOTATATE PET, **D** image from ROVER adaptive thresholding software

**Fig. 2.**
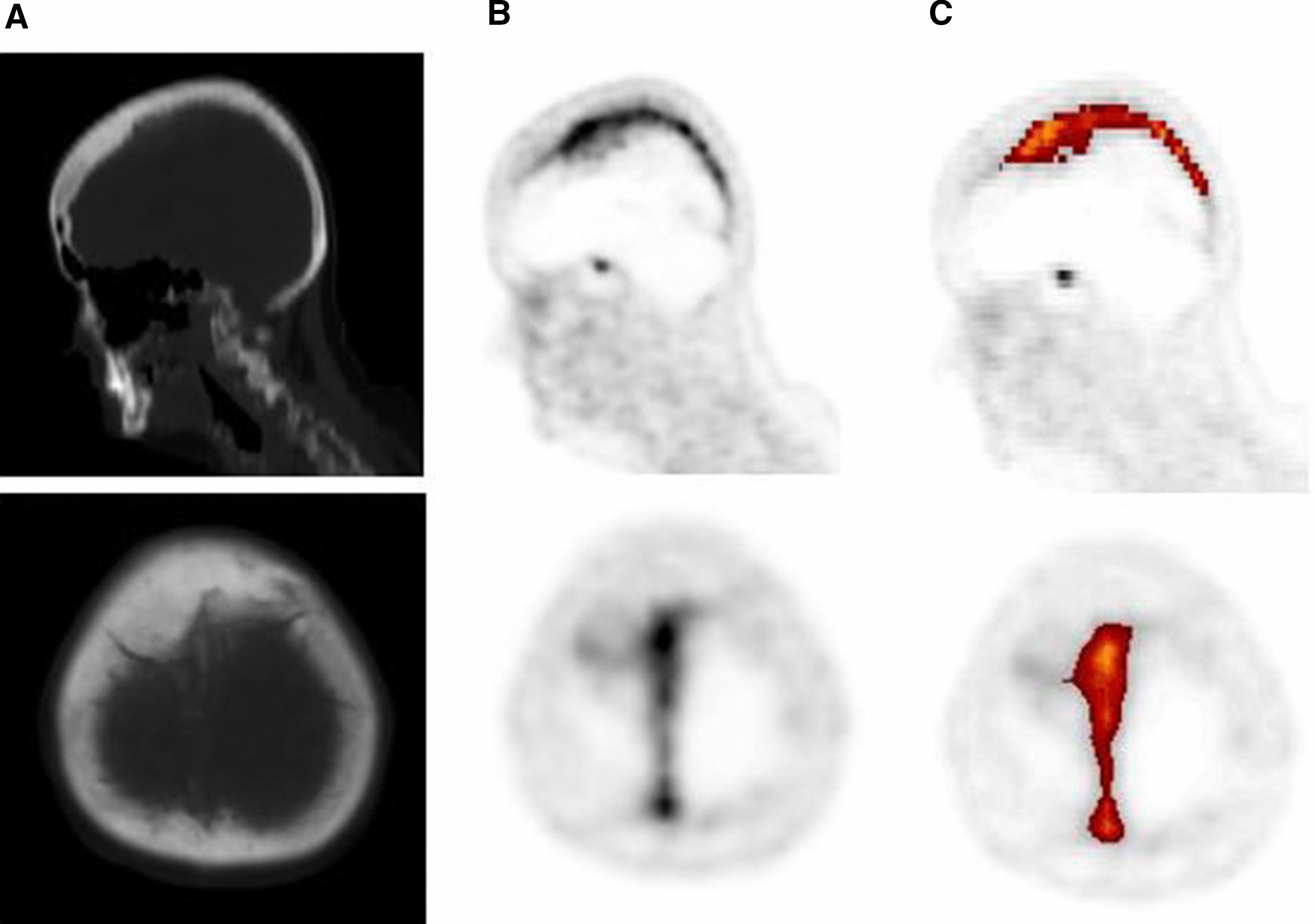
68 Ga-DOTATATE PET highlighting a meningioma with falx involvement. **A** Sagittal and axial CT-scans, **B** Sagittal and axial 68 Ga-DOTATATE PET images. **C** Images from ROVER adaptive thresholding software

**Fig. 3.**
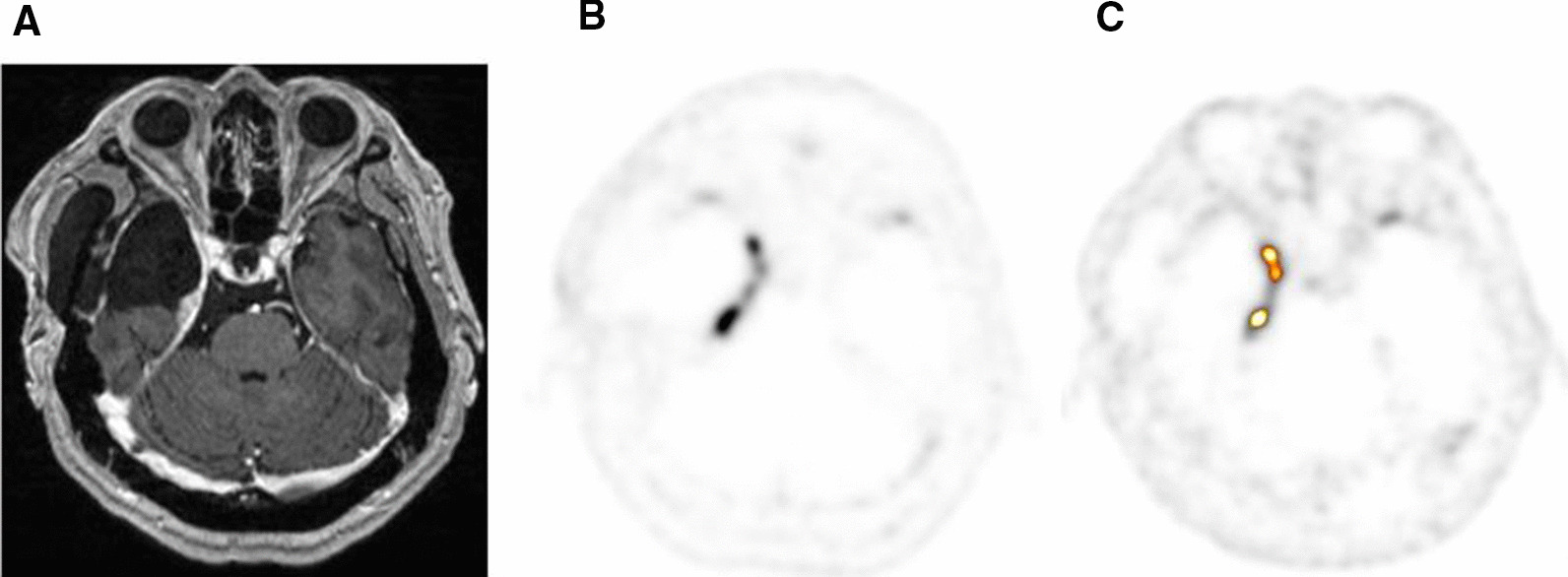
^68^ Ga-DOTATATE PET identifying residual meningioma in the setting of prior resection. **A** T1 post-contrast MRI, **B** ^68^ Ga-DOTATATE PET, C. Image from ROVER adaptive thresholding software

^68^ Ga-DOTATATE PET/CT findings changed clinical management of three patients. An 86 year old male presented with worsening vision and a progressing right temporal lobe/ medial sphenoid wing meningioma. Initially the patient was planned for five fractions of stereotactic RT, however conventional fractionation was pursued when planning ^68^ Ga-DOTATATE PET/CT highlighted disease compressing the right optic nerve (Fig. 4A). Planning ^68^ Ga-DOTATATE PET/CT excluded the diagnosis of meningioma in 46-year old female with a history of treated Stage 3A breast cancer who presented with subacute onset of ataxia and falls and was found to have a 2.6 cm homogenously enhancing, extra-axial, dural-based mass originating from the right cerebellar tentorium on MRI. ^68^ Ga-DOTATATE PET/CT was performed and this lesion demonstrated mild dotatate uptake, SUVmax = 4.1 (Fig. 4B). In light of the patient’s history of breast cancer and less than expected dotatate uptake, multi-disciplinary consensus deemed the lesion more likely a brain metastasis than a meningioma. The patient was treated with radiosurgery in one fraction of 18 Gy. The patient developed radiation necrosis and underwent surgical biopsy prior to laser interstitial thermal therapy. Pathology revealed substantial reactive gliosis but no viable tumor, in support of the diagnosis of brain metastasis as it is uncommon for meningiomas to cause large areas of reactive gliosis in the absence of brain invasion.

^68^ Ga -DOTATATE PET/CT showed additional osseous involvement not well appreciated on MRI in four patients with base of skull tumors and in one patient with a parietal calvarial lesion (Fig. 1). ^68^ Ga -DOTATATE PET/CT better emphasized the extent of falx involvement in five patients (26%). ^68^ Ga-DOTATATE PET/CT supported a diagnosis of meningioma in one patient who presented with headaches and was found to have diffuse enhancement and thickening of the superior sagittal sinus and falx on MRI, but had previously undergone a non-diagnostic biopsy. This lesion was avid on ^68^ Ga-DOTATATE PET/CT (Fig. 2). In the setting of prior resection, ^68^ Ga-DOTATATE PET/CT identified residual tumor after surgical resection to include in radiation target volumes (Fig. 3). Additionally, ^68^ Ga-DOTATATE PET/CT showed clinically asymptomatic satellite lesions in three patients (18%) that had not been previously identified based on MRI.

**Fig. 4:**
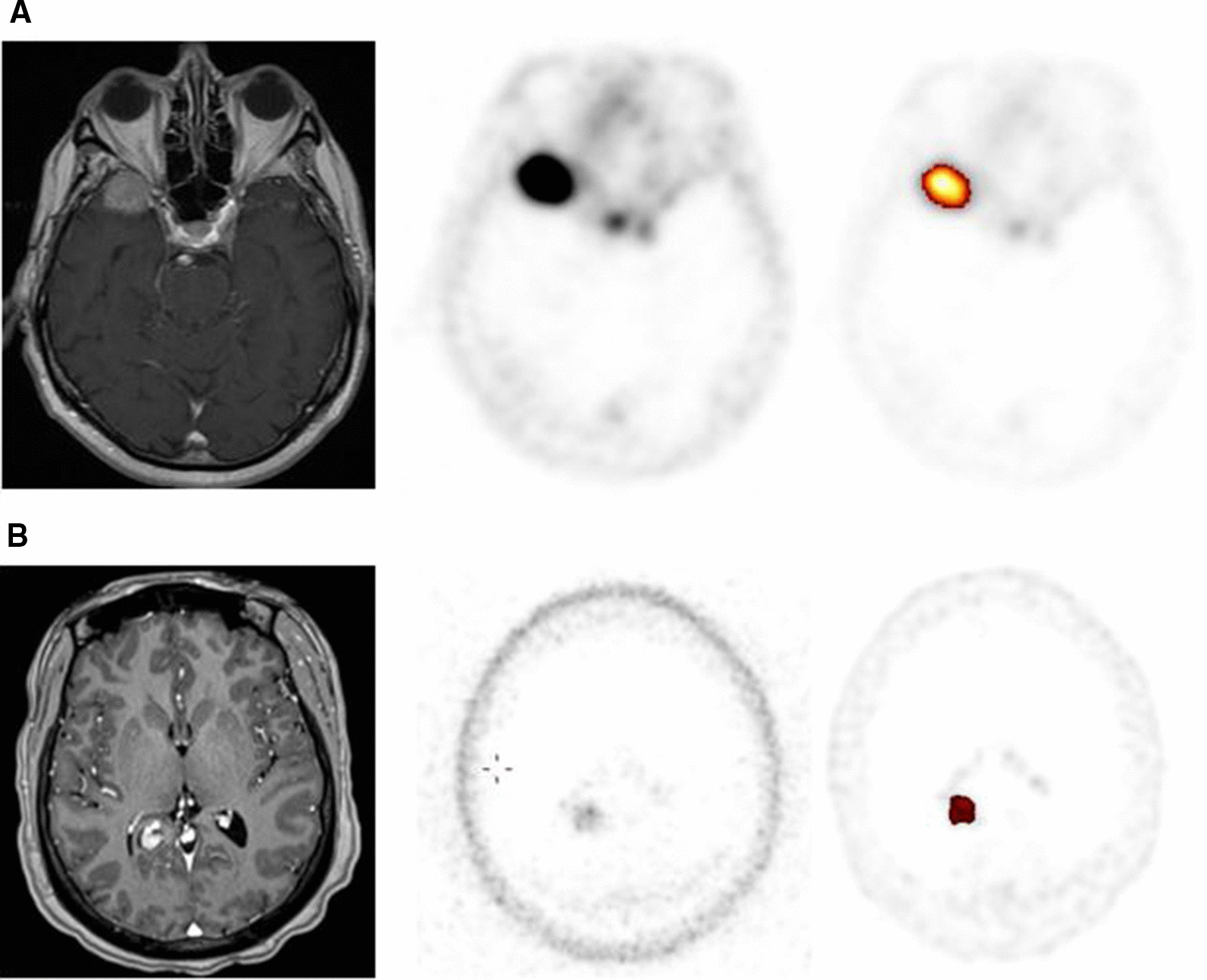
Select patient cases for which radiation planning 68 Ga-DOTATATE PET changed clinical management. **A** Imaging demonstrating proximity of right medial sphenoid wing meningioma to the optic nerve. MRI, 68 Ga-DOTATATE PET and image from ROVER adaptive thresholding software. **B** Imaging demonstrating cerebellar tentorial lesion with mild dotatate uptake (SUV max = 4.1) favoring diagnosis of brain metastasis and treatment with radiosurgery in patient with remote history of breast cancer. MRI, 68 Ga-DOTATATE PET and image from ROVER adaptive thresholding software

**Fig. 5:**
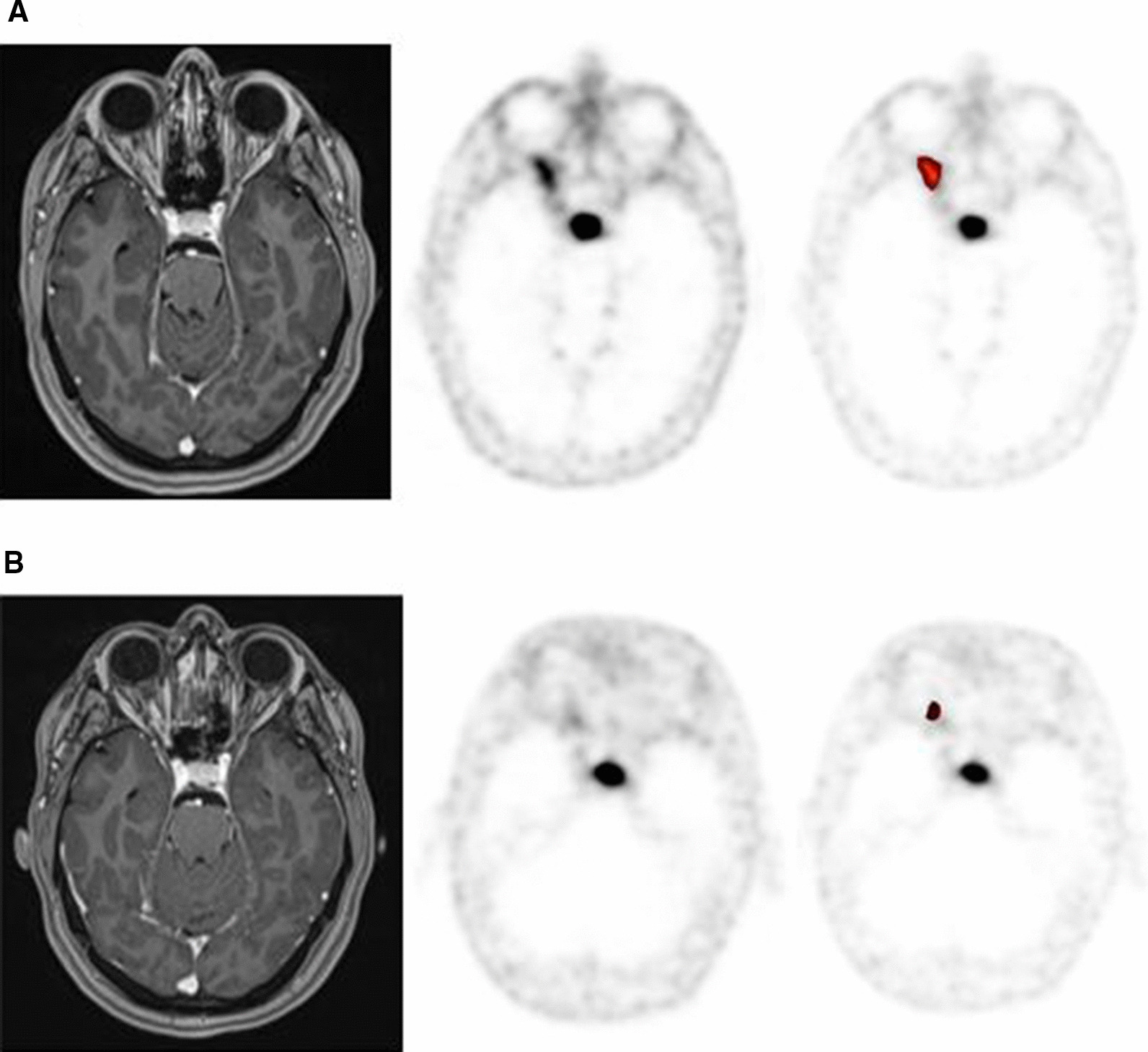
Optic nerve sheath meningioma. **A** Pre-RT imaging: T1 post-contrast MRI, Pre-treatment 68 Ga-DOTATATE PET, SUV Max = 6.3, image from ROVER adaptive thresholding software. **B** Three month post-RT PET-MR imaging: T1 post-contrast MRI, 68 Ga-DOTATATE PET/CT, SUV Max = 3.8, image from ROVER adaptive thresholding software

Planning ^68^ Ga-DOTATATE PET/CT supported a diagnosis of suspected optic nerve sheath meningioma. The patient, a 22-year old female with a family history of sarcoidosis, presented with an 8-month history of progressive decreased vision in her right eye. Ophthalmologic exam revealed optic disc pallor. Post-contrast MRI demonstrated a T1 hyperintense and T2 isointense lesion involving the intraocular right optic nerve extending into the intracanalicular segment. Differential diagnoses included optic nerve sheath meningioma, lymphoma, sarcoidosis, infection, inflammatory process and orbital pseudotumor. CT without contrast did not display classic calcifications encasing the optic nerve [14]. Neurosurgical evaluation deemed biopsy unsafe. ^68^ Ga-DOTATATE PET/CT established asymmetric increased dotatate avidity (SUV = 6.3) corresponding to the known lesion, supporting the diagnosis of optic nerve sheath meningioma. The patient was treated with definitive PBSPT to a dose of 50.4 Gy in 28 fractions. There was no detectable change on post-RT MRI, SUVmax on ^68^ Ga-DOTATATE PET/CT decreased 40% from 6.3 to 3.8 after RT (Fig. 5). At three months the patient reported improved right sided vision and recent ophthalmologic exam documented visual improvement.

### Post-treatment imaging

**Fig. 6:**
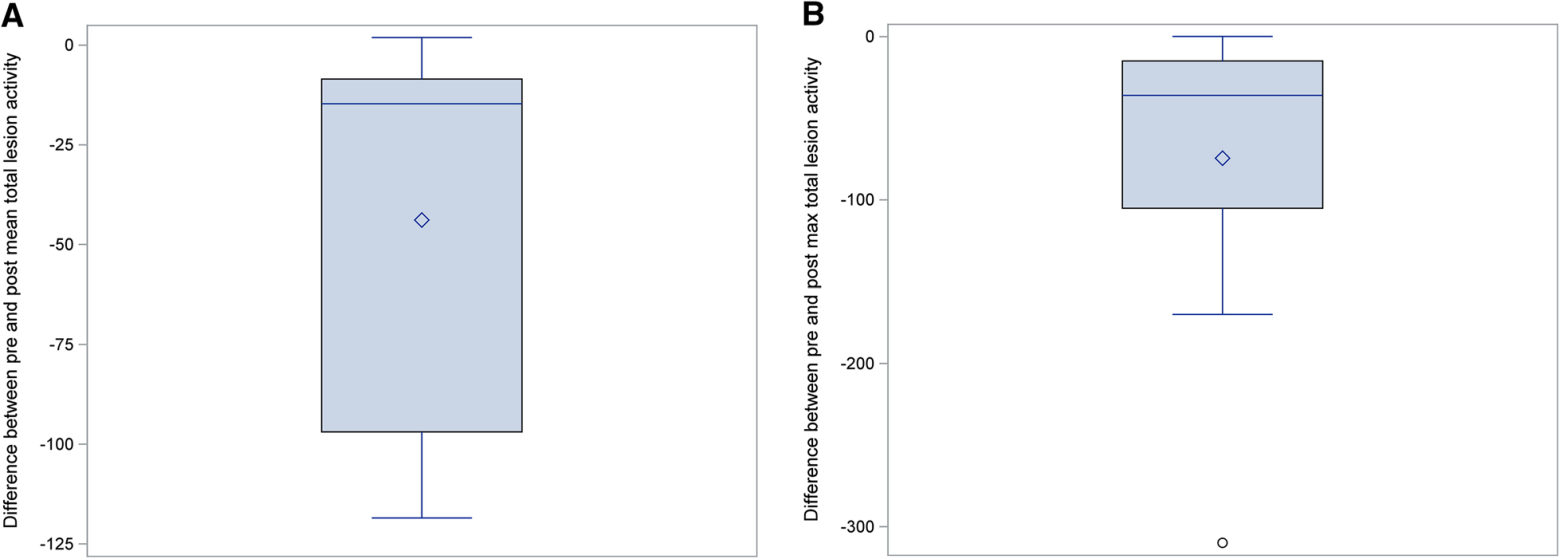
Box plot: post-RT mean (**A**) and max (**B**) total lesion activity

In the subset of patients who underwent both planning and follow-up ^68^ Ga-DOTATATE PET/CT (n = 10), mean total lesion activity decreased 14.7% (median), from pre to post-RT ^68^ Ga-DOTATATE PET [range 97–8.5% (25–75%), S = − 26.5, *p* = 0.0039]. Max total lesion activity decreased 36% (median) over the same period [range 105–15% (25–75%), S = − 26.5 *p* = 0.0039]. Figure 6. This decrease in mean and max total lesion activity was observed in 9 of 10 patients (Table 2). Inability to detect the same relationship in the case of the optic nerve sheath meningioma is likely attributable to the lesion’s small size and background activity from pot-treatment changes. Total mean and max activity of the adjacent medial rectus (0.1, 0.2) and contralateral normal left optic nerve were (0.1, 0.1) when measured utilizing the ROVER software.

**Table 2:** Mean and max total lesion activity pre and post radiation treatment measured by ROVER adaptive threshholding software

Tumor location	Pre-RT PET mean total lesion activity	Range	Post-RT PET mean total lesion activity	Range	Pre-RT PET max total lesion activity	Range	Post-RT PET max total lesion activity	Range
Overall population (Median)	61.2	5.3–550.7	42.9	5.6–440.6	79.2	8.5–600.9	51.2	6.0–430.9
Individual patient data								
Base of skull (parasellar)	550.7		440.6		600.9		430.9	
Base of skull (cavernous sinus)	68.6		54.5		85.9		65.1	
Base of skull (spenoid)	159.1		40.6		377.3		67.5	
Base of skull (sphenoid)	13.2		5.6		11.8		6	
Falx	47.6		32.3		48.2		33.3	
Falx	167.1		111.6		130.9		85.4	
Falx	53.7		45.2		70.4		43.9	
Brain convexity	38.5		24.4		72.4		25.9	
Optic nerve	5.3		7.2		8.5		8.6	
Cerebellar pontine angle	148.1		51.2		163.5		58.4	

In contrast, meningioma volumes were stable on the corresponding pre and post-RT MRI images per RECIST criteria and corroborated by Wilcoxon signed rank test which showed no significant difference in mengingioma volume comparing these two timepoints (S = − 3, *p* = 0.7422). One patient was determined to have radiation necrosis on follow-up MRI and another was unable to undergo a follow-up MRI due to the presence of a pacemaker. Clinical improvement in presenting neurologic symptoms was observed in 7 patients (70%).

## Discussion

In this study, we evaluated the potential role of ^68^ Ga-DOTATATE PET/CT in addition to MRI for both radiation planning and post-treatment surveillance of meningiomas. This work provides novel findings characterizing the radiotherapeutic responses using ^68^ Ga-DOTATATE PET/CT three months after completion of radiotherapy. Using PET-based assessments, the majority of patients in this series (90%) responded to RT, mean total lesion activity decreased by 15% and max total lesion activity decreased by 36% and most patients experienced improvement in their presenting neurologic symptoms. At the same time point, MRIs noted stable disease.

Importantly, ^68^ Ga-DOTATATE PET/CT modified the clinical management of three patients. ^68^ Ga-DOTATATE PET/CT supported a diagnosis of optic nerve sheath meningioma and conversely, in another case ruled out a diagnosis of meningioma in favor of a brain metastasis. In yet another patient, ^68^ Ga-DOTATATE PET/CT illustrated the extent of optic nerve involvement, prompting treatment with conventional fractionation rather than SRS. When ^68^ Ga-DOTATATE PET/CT images were incorporated alongside MRI and CT for contouring, we found our clinical target volumes to be slightly larger because ^68^ Ga-DOTATATE PET/CT highlighted areas of involvement (base of skull, osseous structures, falx) not definitively visualized on conventional imaging. In the postoperative setting, ^68^ Ga-DOTATATE PET/CT helped distinguish residual disease from post-operative changes. Additionally ^68^ Ga-DOTATATE PET/CT can be helpful when patients have pacemarkers or ICDs that are not compatible with MRI.

Recently, the benefits of including PET in radiotherapy planning were supported in a study of 339 meningiomas by Kessel et al.. They utilized PET in planning and target volume definition for radiation post-operatively or at recurrence and demonstrated significantly improved local control [15]. They hypothesized that this resulted from PET’s ability to improve detection of tumor cells and better distinguish between healthy and meningioma tissue [15].

Prior studies evaluating alternative imaging for surveillance of meningiomas, highlighted in the RANO/PET Group’s recent report [16], utilized older techniques or radiotracers targeting different mechanisms. Gudjonsson and co-investigators assessed the utility of pre-and post-treatment ^11^C-L-methionine PET in a series of nineteen patients undergoing fractionated stereotactic proton radiotherapy for meningiomas [17]. Thirty-six months after delivering 24 Gy in 4 consecutive daily fractions the investigators observed nearly a 20% reduction in the radioactivity ratio of tumor to normal brain from pre to post treatment PET but were unable to appreciate significant volume reductions on MRI and suggested that ^11^C-L-methionine PET could provide an earlier assessment of treatment response than MRI [17]. ^11^C-L-methionine PET utilizes a radiolabeled amino acid analogue for which meningiomas show increased uptake. However, ^68^ Ga-DOTATATE PET/CT allows for even better contrast between tumor and background brain than ^11^C-L-methionine PET [16].

Nicolato and colleagues utilized somatostatin receptor imaging to evaluate treatment response in 20 patients with WHO grade I symptomatic cavernous sinus meningiomas. 7–12 months after SRS, cranial nerve deficits improved in the majority (75%) of patients, however MRI at this timepoint demonstrated decrease in tumor volume in only 15% of patients, similar to the pattern observed in our series. Utilizing SPECT with octreotide, neurologic responders demonstrated a more significant decrease in receptor activity at the same timepoint after SRS than nonresponders (*p* < 0.05) [18].

Meningiomas regress slowly after radiation and consequently follow-up of these tumors requires serial MRIs. This can be burdensome for patients and costly to the healthcare system. For the same reasons, shorter-term imaging endpoints in clinical trials with these tumors can be difficult to measure. Additionally, meningiomas are often located adjacent to cranial nerves and critical brain structures, therefore only a small volumetric increase could cause symptoms. For these reasons, an imaging modality like ^68^ Ga-DOTATATE PET/CT may be an earlier indicator of positive treatment response and thus could be of considerable benefit.

The recently published NETTER-1 trial demonstrated dramatically improved progression free survival adding ^177^Lu-Dotatate to octreotide therapy in patients with somatostatin-receptor-positive midgut neuroendocrine tumors [19]. These results revived interest in peptide receptor radionuclide therapy for refractory meningiomas. A single arm phase II investigation administering Lutathera (^177^Lu-DOTATATE) to patients with refractory or high-risk meningiomas is currently being conducted. This study will assess tumor response 6 months post drug initiation with ^68^ Ga-DOTATATE PET/MRI (ClinicalTrials.gov identifier: NCT03971461). High expression of SSTR2 has been shown to correlate with faster tumor growth rates on MRI in WHO grade I and II meningiomas [20]. Further investigation into the utility of ^68^ Ga-DOTATATE PET/CT to identify non-responders to radiotherapy and potential candidates for peptide receptor radionuclide therapy is warranted. ^68^ Ga-DOTATATE PET/CT could also prove useful in the setting of initial observation or surveillance of recurrent meningiomas. SUVmax could help distinguish active, recurrent meningioma from scar tissue and prior treatment induced changes, a distinction that can be challenging based on CT or MRI alone [16].

This series has inherent limitations. This was a single retrospective institution analysis with a limited sample size. Separate traditional MRI based CTV volumes and ^68^ Ga-DOTATATE PET/CT based CTV volumes were not contoured precluding quantification of the difference in treatment volumes generated with each imaging modality. Histopathologic confirmation of the change in concentration of SSTR receptors after radiotherapy was not performed. The median follow-up (3 months) for the cohort was short. Prospective studies utilizing a systematic approach to measuring longer-term changes with ^68^ Ga-DOTATATE PET/CT are needed to validate that decreased uptake of dotatate after radiotherapy is associated with high tumor control rates and the prognostic value of the degree and timing of response measured on ^68^ Ga-DOTATATE PET/CT imaging. Functional imaging ^68^ Ga-DOTATATE PET/CT has inherently inferior spacial resolution than MRI. Accurate assessment of response can be limited by the small size of the lesion as demonstrated in the case of the optic nerve lesion in our population. It’s ability to identify areas not well seen on MRI make it a useful adjunct rather than replacement imaging modality.

## Conclusions

Taken together, our findings contribute to the growing body of evidence supporting the use ^68^ Ga-DOTATATE PET/CT as an adjunctive imaging modality to help aid in the diagnosis and radiation treatment planning of meningiomas. This study represents one of the first series evaluating the response of meningiomas to radiotherapy utilizing ^68^ Ga-DOTATATE PET/CT. In this population, meningioma uptake of the SSTR2 ligand ^68^ Ga-DOTATATE decreased by three months after RT, even in absence of objective MRI response. Future prospective studies are necessary to determine the prognostic significance of ^68^ Ga -DOTATATE PET/CT post-treatment imaging changes and to characterize molecular profiles of PET responders vs. non-responders.

## Data Availability

Data are available upon request to the corresponding author.

## Abbreviations

GTV: Gross tumor volume
ICD: Implantable cardioverter defibrillator
IMRT: Intensity modulated radiation therapy
MRI: Magnetic resonance imaging
PBSPT: Pencil beam scanning proton therapy
PET: Positron emmission tomography
PVC: Partial volume corrected
RECIST: Response evaluation criteria in solid tumours
RT: Radiation therapy
SRS: Stereotactic radiosurgery
SSTR2: Somatostatin receptor-2
SUV: Standardized uptake value
TLA: Total lesion activity
WHO: World Health Organization

## References

[1] Ostrom QT, Gittleman H, Truitt G, Boscia A, Kruchko C, Barnholtz-Sloan JS (2018). CBTRUS statistical report: primary brain and other central nervous system tumors diagnosed in the United States in 2011–2015. Neuro Oncol.

[2] Reubi JC, Schaer JC, Waser B, Mengod G (1994). Expression and localization of somatostatin receptor SSTR1, SSTR2, and SSTR3 messenger RNAs in primary human tumors using in situ hybridization. Cancer Res.

[3] Graillon T, Romano D, Defilles C (2017). Octreotide therapy in meningiomas: in vitro study, clinical correlation, and literature review. J Neurosurg.

[4] Klutmann S, Bohuslavizki KH, Brenner W (1998). Somatostatin receptor scintigraphy in postsurgical follow-up examinations of meningioma. J Nucl Med.

[5] Rachinger W, Stoecklein VM, Terpolilli NA (2015). Increased 68Ga-DOTATATE uptake in PET imaging discriminates meningioma and tumor-free tissue. J Nucl Med.

[6] Afshar-Oromieh A, Giesel FL, Linhart HG (2012). Detection of cranial meningiomas: comparison of68Ga-DOTATOC PET/CT and contrast-enhanced MRI. Eur J Nucl Med Mol Imaging.

[7] Klingenstein A, Haug AR, Miller C, Hintschich C (2015). Ga-68-DOTA-TATE PET/CT for discrimination of tumors of the optic pathway. Orbit.

[8] Kunz WG, Jungblut LM, Kazmierczak PM (2017). Improved detection of transosseous meningiomas using^68^Ga-DOTATATE PET/CT compared with contrast-enhanced MRI. J Nucl Med.

[9] Graf R, Nyuyki F, Steffen IG (2013). Contribution of 68Ga-DOTATOC PET/CT to target volume delineation of skull base meningiomas treated with stereotactic radiation therapy. Int J Radiat Oncol Biol Phys.

[10] Milker-Zabel S, Zabel-du Bois A, Henze M (2006). Improved target volume definition for fractionated stereotactic radiotherapy in patients with intracranial meningiomas by correlation of CT, MRI, and [68Ga]-DOTATOC-PET. Int J Radiat Oncol Biol Phys.

[11] Gehler B, Paulsen F, Öksüz MT (2009). [68Ga]-DOTATOC-PET/CT for meningioma IMRT treatment planning. Radiat Oncol.

[12] Nyuyki F, Plotkin M, Graf R (2010). Potential impact of 68Ga-DOTATOC PET/CT on stereotactic radiotherapy planning of meningiomas. Eur J Nucl Med Mol Imaging.

[13] Therasse P, Arbuck SG, Eisenhauer EA (2000). New guidelines to evaluate the response to treatment. J Natl Cancer Inst.

[14] Ortiz O, Schochet SS, Kotzan JM, Kostick D (1996). Radiologic-pathologic correlation meningioma of the optic nerve sheath. Am J Neuroradiol.

[15] Kessell K, Weber W, Yakushev I, Fischer H, Voglhuber T, Diehl C, Straub C, Zimmer C, Wiestler B, Gempt J, Meyer BCS (2019). Integration of PET-imaging Into radiotherapy treatment planning for low-grade meningiomas improves outcome. Eur J Nucl Med Mol Imaging.

[16] Galldiks N, Albert NL, Sommerauer M (2017). PET imaging in patients with meningioma—report of the RANO/PET group. Neuro Oncol.

[17] Gudjonsson O, Blomquist E, Lilja A, Ericson H, Bergstrom M, Nyberg G (2000). Evaluation of the effect of high-energy proton irradiation treatment on meningiomas by means of 11C-L-methioine PET. Eur J Nucl Med.

[18] Nicolato A, Giorgetti P, Foroni R (2005). Gamma knife radiosurgery in skull base meningiomas: A possible relationship between somatostatin receptor decrease and early neurological improvement without tumour shrinkage at short-term imaging follow-up. Acta Neurochir (Wien).

[19] Strosberg J, El-Haddad G, Wolin E (2017). Phase 3 trial of 177 Lu-Dotatate for midgut neuroendocrine tumors. N Engl J Med.

[20] Sommerauer M, Burkhardt JK, Frontzek K (2016). 68Gallium-DOTATATE PET in meningioma: a reliable predictor of tumor growth rate?. Neuro Oncol.

